# A Plea for the Extended Use of Atomised Drugs

**Published:** 1908-05-02

**Authors:** Edwin Ash

**Affiliations:** 30 Harley Street, W.


### A PLEA FOR THE EXTENDED USE OF ATOMISED DRUGS.

(*To the Editor of* The Hospital.)

Sir,—The only common use to which atomisers are put at the present day is in the treatment of "catarrhs" of the nose and throat; but there is no doubt that there is a much more extensive field for the use of atomised drugs than is generally acknowledged. I think the most serviceable way of obtaining atomisation is by means of the steam-spray; this method was brought forward by Siegle many years ago, but the useful apparatus which bears his name seems to have fallen into disuse. It is my opinion that the steam-spray atomiser has a very useful field in the treatment of local affections apart from diseases of the nose and throat, for by its use we can direct a constant stream of fine particles of any required drug on to the site of local disease, and at the same time the condensation of the steam gives us a means of constantly washing away discharges and their contained organisms. Thus we have two most important actions going on simultaneously, namely, the close contact of the finely-divided drug with the diseased tissue and an important cleansing action due to the stream of condensing water-vapour.

The ordinary forms of steam-spray atomiser such as that devised by Siegle are primarily intended for inhalation purposes, and so have no provision for directing the stream of atomised drug on to any particular spot; but by means of a simple modification the steam-spray atomiser may be efficiently used for this purpose. This modification consists in having the two atomiser-tubes (the essential part of the whole apparatus) connected to the boiler and to the receptacle containing the drug to be atomised by long, flexible rubber tubes, so that by a convenient handle the atomising tubes, and consequently the mixed stream of atomised drug and steam, can be directly applied to any particular accessible point for any length of time. This modification enables us to deal with superficial lesions, such as obstinate ulcers, badly-healing wounds, septic skin lesions, and similar affections, besides giving us a more efficient spray for accessible regions of the nose and throat.

I am, Sir, yours faithfully,

30 Harley Street, W., April 20. Edwin Ash.

